# Investigation of the mechanisms for wireless nerve stimulation without active electrodes

**DOI:** 10.1002/bem.22486

**Published:** 2023-11-01

**Authors:** Luke A. Smith, Jaedon D. Bem, Xiaojing Lv, Antonio Lauto, Ashour Sliow, Zhiyuan Ma, David A. Mahns, Carolyn Berryman, Mark R. Hutchinson, Christophe Fumeaux, Giuseppe C. Tettamanzi

**Affiliations:** ^1^ School of Electrical and Electronic Engineering University of Adelaide Adelaide Australia; ^2^ School of Science Western Sydney University Penrith New South Wales Australia; ^3^ School of Medicine Western Sydney University Penrith New South Wales Australia; ^4^ School of Biomedicine University of Adelaide Adelaide South Australia Australia; ^5^ Adelaide Medical School, Institute of Photonics and Advanced Sensing University of Adelaide Adelaide South Australia Australia; ^6^ Discipline of Materials Engineering, School of Chemical Engineering University of Adelaide Adelaide South Australia Australia

**Keywords:** computational electromagnetics, electromagnetic stimulation, graft‐antenna, magnetostatics, nerve stimulation, neural activation

## Abstract

Electric‐field stimulation of neuronal activity can be used to improve the speed of regeneration for severed and damaged nerves. Most techniques, however, require invasive electronic circuitry which can be uncomfortable for the patient and can damage surrounding tissue. A recently suggested technique uses a graft‐antenna—a metal ring wrapped around the damaged nerve—powered by an external magnetic stimulation device. This technique requires no electrodes and internal circuitry with leads across the skin boundary or internal power, since all power is provided wirelessly. This paper examines the microscopic basic mechanisms that allow the magnetic stimulation device to cause neural activation via the graft‐antenna. A computational model of the system was created and used to find that under magnetic stimulation, diverging electric fields appear at the metal ring's edges. If the magnetic stimulation is sufficient, the gradients of these fields can trigger neural activation in the nerve. In‐vivo measurements were also performed on rat sciatic nerves to support the modeling finding that direct contact between the antenna and the nerve ensures neural activation given sufficient magnetic stimulation. Simulations also showed that the presence of a thin gap between the graft‐antenna and the nerve does not preclude neural activation but does reduce its efficacy.

## Highlights

- This newly accepted paper describes and provides model insights regarding one of the first examples of wireless stimulation and repair of peripheral nerves. To outline the relevance of this work it is important to state that the wireless *stimulation and repair of peripheral nerves research performed by some of the authors of this paper* has also been already discussed by the very prestigious MIT Technical Review (https://www.technologyreview.com/2018/07/23/240392/nerves-repaired-using-bioscaffold-fitted-with-radio-antenna/) as very relevant.

## 1 INTRODUCTION

Nerve damage and associated chronic pain are debilitating conditions which weigh heavily on the individual and burden society. Chronic pain due to nerve damage, by definition, does not resolve in the expected timeframe for most injuries (<3 months). People with this condition are at increased risk of depression and, for want of more effective pain relief options, are often prescribed opioids, adding serious side effects such as addiction and death to the burden of managing chronic pain (Aamir et al., 2020; Bouhassira et al., 2008; Colloca et al., 2017; Health Direct, 2018; Kawai et al., 2017; Torrance et al., 2006; de Moraes Vieira et al., 2012). The total cost of chronic pain for society, in the United States during 2008, is over $600 billion, including $300 billion for health care and $335 billion for loss of productivity (Gaskin & Richard, 2012; Howe & Sullivan, 2014; Kawai et al., 2017).

A treatment for nerve damage and chronic pain comes in the form of repeated electrical stimulation. It helps accelerate nerve repair which used to be a time‐critical activity with a low success rate (Borgens, 1999; Lal et al., 2008; Patel & Poo, 1982). Transcutaneous Electric Nerve Stimulation (TENS), for example, is an electrical stimulation method noninvasive to the nerve, however, there exists conflicting evidence surrounding its efficacy in pain relief (Magrinelli et al., 2013). Electrical stimulation can also be applied with implants that require invasive intervention. A power source and internal circuitry must be embedded near the site of repair, a process that can damage and scar the surrounding tissue, thus hampering recovery. Moreover, the risk of surgical complications is further raised as the circuitry may eventually be removed from the body. A novel minimally invasive treatment devised by Lauto and coworkers used a suture‐less graft‐antenna to reconnect the ends of a severed rat sciatic nerve (Sliow et al., 2019).

Although it has been already experimentally demonstrated that repeated electrical stimulation of nerves improves their regenerative ability (Borgens, 1999; Lal et al., 2008; Patel & Poo, 1982), the physical mechanism is still unclear. Recent studies have modelled the TMS‐induced electric field in rat brains (Koponen et al., 2020), however how specifically the electromagnetic fields interact with the metal ring in the graft‐antenna to cause neural activation, needs also exploration. Furthermore, when a loosely attached, copper toroidal ring was used instead of the standard ribbon ring, an interesting phenomenon was observed in graft‐antennas. In fact, gaps of approximately 100 µm between the ring and the nerve prevented neural activation, even under the highest level of magnetic stimulation intensity available to the magnetic stimulation device (Sliow et al., 2019). From this, it was postulated that close contact between the nerve and the toroidal ring (and hence any graft‐antenna) was necessary for neural activation. In this study, we measured in vivo action potentials triggered in rat sciatic nerves coupled to graft antenna to validate previous findings (Sliow et al., 2019) and develop a computational model (e.g. Sim4Life Application and Support Team, 2020b) to fit the experimental data. We focused in particular on the “contact effect” regarding the cause, the level of acceptable separation, and the effective choice of cross‐sectional geometry of the ring. It was also determined that the electrical stimulation of the nerve was not due to the fields induced by the magnetic stimulation device alone or due to currents flowing from the graft‐antenna into the nerve. These investigations led to the hypothesis that the neural response was due to electric fields generated tangentially to the graft‐antenna at the interface with the nerve (Sliow et al., 2019), which needs further verification with the aid of electromagnetic modeling and full‐wave simulation.

In this work, we aimed to investigate the most likely mechanisms allowing the graft‐antenna system to activate peripheral nerves and establish whether or not contact between the nerve and the graft‐antenna is necessary for triggering action potentials. If contact is not necessary, we aim to further explore the effect that separation distance has on neural activation and determine at what range of distances activation stops.

## 2 MATERIALS AND METHODS

The stimulation mechanism can be explored with the broader concepts of the graft‐antenna's operation; therefore, in view of the inherent variability of biological systems, the considerations are qualitative in nature. This makes the final statements robust to scaling changes in the experiment, such as differences in stimulation intensity, rat size, and relative positions between the magnetic coil and the rat. The analysis was performed in the electromagnetic simulation tool CST 2021 Studio Suite (Dassault Systemes, 2021), where the finite integration technique (FIT) using adaptive hexahedral meshing was adopted to create models of the nerve and ribbon ring graft‐antenna and, on one occasion, the nerve and a toroidal ring. All models were stimulated by a model magnetic stimulation device based on the one used in (Sliow et al., 2019), and the electric field gradients generated under the surface of the nerve were examined. Unless otherwise stated, all graphs are generated using results collected from the CST environment. A supplementary and more complex nerve model created in the Sim4Life v5.0 environment (Sim4Life, 2020a) was used to cross‐validate these results in a more realistic setting. From these models, it was found that the strong electric fields generated at the edges of the ribbon ring of the graft‐antenna create electric gradients within the nerve when under magnetic stimulation, triggering neural activation.

The analysis was extended by adding a gap between the ribbon ring and the nerve and observing the changes to the electric fields and gradients within the nerve. The graft‐antenna became unable to activate the neurons if the separation implemented by a dielectric layer of low relative permittivity (close to air) is over 100 µm, which was a possible explanation for the contact effect observed in (Sliow et al., 2019). Our modeling showed nonetheless that contact was not necessary for neural activation and microscopic gaps still allowed activation to occur. To further address these questions, we have also performed some measurements in vivo on rats (*n* = 9) to assess the contact effect on nerve stimulation. This paper concludes by proposing pathways for future research.

### 2.1 Equivalent modeling for magnetic stimulation device

The magnetic stimulation device used was a Transcranial Magnetic Stimulation (TMS) Device, which is a common medical instrument that generates high‐intensity electromagnetic pulses for non‐invasive stimulation of nervous tissue. It is typically used for the treatment of depression when standard treatments are shown to have limited effectiveness. In this study, the TMS was not used to stimulate the brain but was instead used to stimulate peripheral nerves.

While TMS devices come in many shapes, sizes, and specifications, the basic design is a current pulse generator connected to a conductive coil. When current is pulsed through this coil (usually as a biphasic or monophasic waveform (Hovey & Jalinous, 2006; Nieminen et al., 2015)), a magnetic field is generated of the order of approximately 0.1 T. This oscillating magnetic field then induces an oscillating electric field which, if correctly aligned, can trigger action potentials within neurons. Usually, this occurs within shallow brain tissue, but in this case, it is used in conjunction with the graft‐antenna to trigger action potentials in peripheral nerves and facilitate axon regeneration. In the rest of this work, we will refer to the stimulation device more generally as a magnetic coil or magnetic stimulation device.

**Figure 1:**
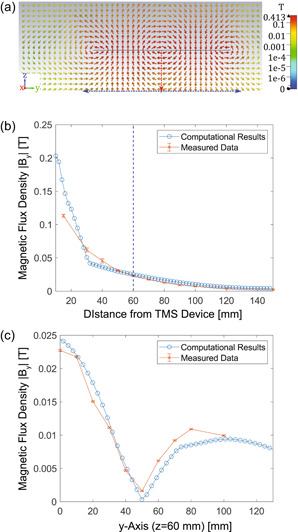
Magnetic vector fields calculated by the model magnetic coil compared against experimental measurements. (a) Side‐on view of the magnetic vector field generated by the model coil with a 1 kA current. The black line indicating the transverse magnetic coil spans 180 mm through the centre as detailed in Figure 9. The red arrow shows the measurement axis for (b), and the blue arrow 60 mm below the magnetic coil shows the measurement axis for (c); (b) The absolute magnitudes of the magnetic flux density, *B*_*y*_, measured along the *z*‐axis. At around 30 mm, near‐field effects and differences in the magnetic coil structures cause the computational measurements to vary from the experimental measurements (Salinas et al., 2007). The blue dashed line highlights where (c) is measured; (c) The absolute magnitudes of the magnetic flux density, *B*_*y*_, measured along the *y*‐axis, 60 mm below the magnetic coil. Measurements are taken from the simulation software and by experimental verification using a Hall effect probe. For the fields to match, a current of approximately 1 kA was needed.

The magnetic coil used in previous reports (Sliow et al., 2019) is a Figure 8 coil, consisting of two connected coils wound in opposite directions. The magnetic fields generated by these coils superimpose directly below the centre of the device, creating a strong magnetic field directed parallel to its long axis, as seen in Figure 1. Depending on the shape and design of the magnetic coil, it is possible to modify the stimulation depth and focality of the produced fields. Magnetic stimulation has the advantage of stimulating superficial nerves to a significantly lesser degree than electrical stimulation, avoiding or reducing the uncomfortable tickling sensations in patients (Colella et al., 2023). Another advantage of magnetic stimulation devices is that non‐invasive stimulation methods are inherently safer than their invasive counterparts, reducing the risk of infection and further tissue damage or inflammation. The graft‐antenna itself is less invasive than sutured implants and is significantly better than needing an internal power supply.

We created a model magnetic coil using the parameters described in the Supporting Information Section: Magnetic stimulation device characteristics. In order to validate that the model was representative of a real magnetic stimulation device, we performed an experiment where we used a Hall effect probe to measure the magnetic flux density component in the *y*‐axis, *B_y_*, generated along two different measurement axes: the *z*‐axis through the centre of the magnetic coil, and the *y*‐axis 60 mm beneath the coil. The positions of these axes, as well as the field distributions measured along them are shown in Figure 1. These measurements were compared with the magnetic fields calculated by the model coil along these axes. As can be seen, the experimental and computational measurements qualitatively agree with each other with a few differences. Notably, in Figure 1b, the computed and measured fields differ at around 30 mm or less, a result of the approximated coil geometries which strongly affect the near‐field (Salinas et al., 2007). However, beyond a distance of 50 mm from the TMS, the computational and experimental models agree strongly. In order for the magnitudes to be equal at 60 mm, the computational model needed a current of 1 kA.

### 2.2 Nerve model

The most well‐accepted model for the internal ionic behavior of neurons is described in the Hodgkin‐Huxley model (Hodgkin & Huxley, 1952). It considers a node of Ranvier on a neuron to be electrically equivalent to a lumped circuit of parallel, nonlinear resistances and a capacitance, representing the behaviors of the neuron bilipid layer and its embedded ion channels. Under normal conditions, the electrical potential of the neuron remains at a resting level. However, if sufficient external stimulation is provided, whether through an injected current or induced electric potential gradient, then the neuron will fire, creating an action potential.

A common extension to this model is the cable model which approximates an axon's process using a number of these lumped circuits connected in series by resistances (Basser & Roth, 1991; Roth & Basser, 1990). Analysis and simulation of these models has identified that in the case of magnetic stimulation, the initial action potential occurred at the point where the gradient of the electric field along the length of the axon was the greatest. That is, for an axon aligned along the *y*‐axis, the initial action potential will occur at the point where $\frac{{dE}_{x}}{\mathrm{dx}}$ is maximum, provided that the gradient surpasses a given activation threshold. From the H.H. model, we adopt the activation threshold concept and its approximate value of 2 mV/mm^2^ based on experimental measurements (discussed later) and previous literature (Kosta et al., 2020, 2019) which will be used to qualitatively establish the ability for neural activation to occur.

Modeling the nerve tissue and surrounding tissue is another issue which needs to be addressed. Results from Pisa et al. (2014). found that the best trade‐off between accuracy and computational expense was achieved by modeling surrounding tissue using an anatomically correct model with heterogeneous but non‐dispersive tissue characteristics. However, their goal was to model an entire wrist containing bone, skin, fat, muscles, and nerves; the nerve itself was modelled as a single homogeneous tube. The purpose of this study was not to create an anatomically detailed model of a nerve but rather to identify why the presence of a graft‐antenna would cause neural activation where an absence would not. Therefore, as long as a qualitative approach is maintained, it is valid to ignore inhomogeneity in the nerve tissues for the sake of reducing computational complexity and providing a clear look into the mechanics of the graft‐antenna free from the clutter caused by inhomogeneous tissue structures.

**Figure 2:**
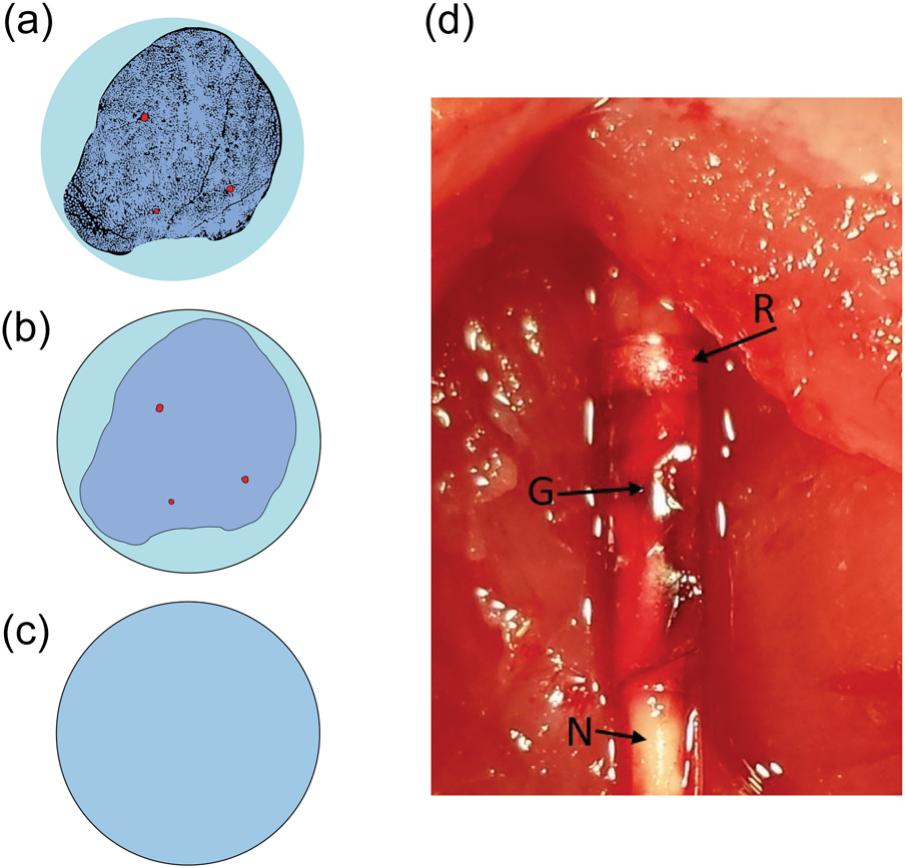
The three nerve models and rat sciatic nerve. Perineurium is cyan, neurons are light blue, and blood vessels are red. (a) The complex model derived directly from the histological cross‐section; (b) Simplified histological model designed in Sim4Life simulation environment; (c) Homogeneous nerve cylinder model; (d) Image of the graft‐antenna bonded in vivo to the sciatic nerve of a rat. The gold ribbon ring (R) that is embedded in the graft‐antenna (G), is in direct contact with the nerve (N).

Three different versions of the nerve were created in the two electromagnetic simulation software environments. These are shown in Figure 2a–c. The first version was an almost direct copy of the histological nerve images provided during rat dis‐section by Sliow et al. (2019). and was computationally infeasible to work with due to the prohibitively high mesh resolution required. The second version of the nerve was a simplified version of the histological cross‐section and contained blood vessels and a nonuniform surface, created using the Sim4Life software environment. The third version of the nerve was a simple homogeneous cylinder. The difference between the results from the second and third models was negligible for our analysis and a more detailed comparison is included in the Supporting Information Section. To reduce computational complexity (a necessity when analysing very small ring thicknesses), the fully homogeneous cylinder model for the nerve was used unless otherwise stated. At this point of our investigation, we gathered in vivo experimental data stimulating the sciatic nerve of rats to validate our model analysis.

### 2.3 In vivo wireless nerve stimulation without active electrodes

A total of nine female Wistar rats weighing 282 ± 8 g were used for the experiments, in compliance with a protocol approved by the ethics committee of Western Sydney University (A10622). The right sciatic nerve (diameter ≈ 1 mm) was exposed according to a standard surgical procedure (See Supporting Information Section: Surgical procedure) and then the animals were randomly allocated to two groups. In the first group (*n* = 3), a copper, toroidal ring with a circular cross sectional‐diameter of 0.10 ± 0.01 mm was wrapped around the nerve, ensuring contact between metal and tissue. In the second group (*n* = 6), a gold, ribbon ring graft‐antenna was laser‐bonded to the sciatic nerve accordingly to a procedure previously published (Sliow et al., 2019) (see Supporting Information Section: Graft‐Antenna Fabrication and Laser Tissue Bonding). The gold, ribbon ring had a rectangular cross‐section (width = 0.8 ± 0.01 mm and thickness of ≈70 nm) which was in direct contact with the nerve after laser bonding (Figure 2d). For reference, the gold ring is always referred to as the “ribbon ring” while the copper ring is referred to as the “toroidal ring.”

The TMS coil used in experiments was positioned 60 mm above the sciatic nerve with the toroidal ring or the graft‐antenna wrapped around the nerve. The coil delivered 1 pulse per second with a duration of ≈350 µs and a current magnitude of ≈ 1.0 kA. In the first group, the sciatic nerve was stimulated uninterruptedly 120 times and the nerve compound action potential (NCAP) was recorded according to a standard protocol (see Supporting Information Section: Electrophysiology Measures). After this procedure, the rats were euthanized. In the second group, the rats survived for 12 weeks to test the ability of the graft‐antenna to effectively trigger action potentials while implanted for a prolonged time interval. Every week the sciatic nerve was stimulated with ten magnetic pulses and the compound muscle action potential (CMAP) of the plantaris muscle, innervated by this nerve, was recorded. At the end of the 12 week‐period the rats of the second group also received a total of 120 pulses. Of note is the fact that magnetic pulses failed in triggering action potentials if the toroidal ring or the ribbon ring graft‐antenna were not positioned around the nerve. These experiments confirmed that the graft‐antenna could trigger neural activation as desired.

## 3 RESULTS

### 3.1 Geometry of baseline nerve stimulation model

**Figure 3:**
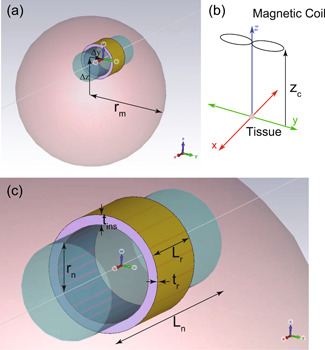
Modeling for wireless nerve stimulation. (a) Zoomed‐out view of the nerve and its relative position within the muscle tissue; (b) Relative position of the TMS coil to the tissue sphere (not to scale); (c) Close‐up view of the nerve with bonded graft‐antenna. The pink sphere represents muscle tissue, and the blue tube is the sciatic nerve. The purple ring is the insulation layer between the nerve and the outermost gold ribbon ring. All solids are homogenised approximations of the respective materials.

For reference purposes, we start with modeling and analysing a baseline scenario of the nerve under magnetic simulation without introducing the graft‐antenna. In practice we are looking here at very near‐field effect at a discontinuity, therefore the performance will not be influenced by the nerve length. The nerve is modelled as a 2.4 mm long cylinder with a radius of 0.5 mm, which is homogeneously filled with a material of effective conductivity 3.11 × 10^−2^ S/m. The muscle tissue enveloping the nerve is modelled as a sphere, of radius 2.5 mm and effective conductivity 3.33 × 10^−1^ S/m. To secure reasonably uniform stimulation from the electric fields within the sphere, the cylindrical nerve is offset by (0, 0.4, 1.2 mm) from the centre of the spheric tissue. The long axis (see Figure 3) of the magnetic coil is transversely oriented to the nerve axis and 60 mm above its centre. To simulate the experimental conditions inherited from the previous work (Sliow et al., 2019), the current peak is 1 kA at an effective operating frequency of 3544 Hz in the wire loop. The gradient of the electric field within the nerve is observed in the simulations as it is the source that initiates the action potential.

On such basis, a gold ribbon ring as the graft‐antenna can be added to the model to investigate the nerve activation. The ring is wrapped around the central segment of the nerve with a thickness of 1 µm and a length of 0.8 mm. The ribbon ring has a distinct and finite layer of insulating material between itself and the nerve with electric properties equivalent to those of air. In practice, this layer could be intentionally manufactured into the antenna design (if desired) or will exist as the result of an imperfect attachment process. The impact of this insulation layer against effective neural activation can be explored by changing the thickness of the insulating layer whilst observing the corresponding electric field (E‐field) gradient along the offset axis. Specifically, configured with different insulation thicknesses of 0, 10, 50, 100, and 500 µm, the model can be simulated with a mesh of approximately 0.5 million tetrahedrons to ensure suitable resolution. The full geometry is shown in Figure 3 with the relevant dimensions contained in Table 1. The material conductivities extracted from the IT'IS database (IT'IS Foundation, 2021) are listed in Table 2. The electric permittivities are irrelevant to the quasi‐magnetostatic solver (see Equation 2 in supplementry information) and are therefore not specified.

**Table 1:** Dimensions for the simulation model.

Geometric parameter	Size [mm]
Spherical muscle radius (*r*_*m*_)	2.5
Cylindrical nerve radius (*r*_*n*_)	0.5
Nerve length (*L*_*n*_)	2.4
Nerve offset (∆_*x*_, ∆_*y*_, ∆_*z*_)	(0, 0.4, 1.2)
Ribbon ring thickness (*t*_*r*_)	1 × 10^−3^
Ribbon ring length (*L*_*r*_)	0.8
Distance from coil (*z*_*c*_)	60

**Table 2:** Conductivities for simulation model.

Material	Conductivity σ [S/m]
Muscle	3.33 × 10^−1^
Nerve	3.11 × 10^−2^
Insulation	1 × 10^−12^
Ribbon ring	4.1 × 10^7^

### 3.2 Insulation effects: estimation of stimulation threshold

**Figure 4:**
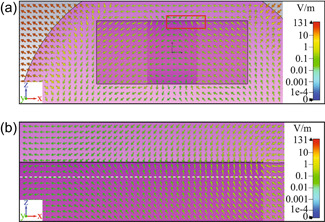
Electric fields distribution around the nerve with gold ribbon ring in contact. (a) Electric fields generated in a broad region of the nerve due to magnetic stimulation; (b) Close‐up view of E‐field vectors within the red rectangular area in (a). The white dash line indicates that the E‐fields are probed 30 µm under the surface of the nerve.

**Figure 5:**
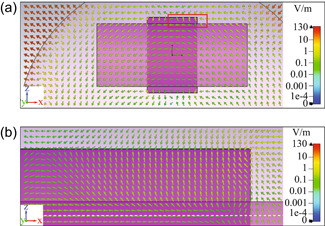
Electric field distribution around the nerve with a 100 µm insulation against the gold ribbon ring. (a) Electric fields generated in a broad region of the nerve due to magnetic stimulation; (b) Close‐up view of E‐field vectors within the red rectangular area in (a). The white dash line indicates that the E‐fields are probed 30 µm under the surface of the nerve.

To illustrate the modeling process, the two cases of no insulation and 100 µm insulation layer are taken as examples. For these two cases, the instantaneous cross‐sectional E‐fields during magnetic stimulation are shown in Figures 4a and 5a. As a result of the spherical geometry, the E‐fields within the muscle tissue tend to flow counter‐clockwise. The E‐fields within the nerve travel along the left‐to‐right direction with slight vertical variations caused by the surrounding tissue, the bounded metallic ring, and other boundary conditions. To see the difference between these two cases, we have to zoom in on the corner of the interface between the ring and the nerve, as shown in Figures 4b and 5b, respectively. Near the metallic ribbon ring, both images demonstrate a significant change (i.e., gradient) of the E‐fields, which could be associated with neural activation. The E‐field vectors in the case of direct contact (Figure 4) transition from horizontal to vertical when approaching the ring's domain from the outside. In contrast, the dynamics for the case with insulation (Figure 5) are much more varied since there is a critical position at which the E‐fields diverge rapidly: some travelling to the left directed parallel to the nerve's surface, whilst others travelling slightly to the right directed perpendicular to the nerve's surface.

Although Figures 4 and 5 provide a visual overview of the instantaneous fields in space, these qualitative representations need to be further investigated. For qualitative considerations, the electric fields are probed 30 µm under the surface of the nerve to avoid irregularities in meshing near boundaries and to capture the thickness of the perineurium. The variation of these fields along the central axis of the nerve is insignificant for both the in‐contact and out‐of‐contact cases.

**Figure 6:**
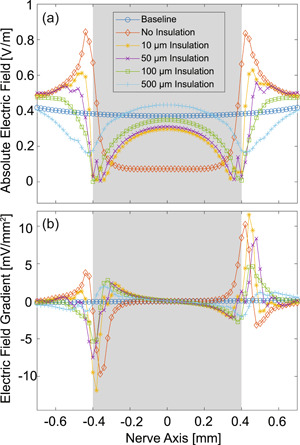
Simulated E‐field strengths and calculated gradients of the ribbon ring along the offset nerve axis 30 µm under the surface of the nerve for different insulation thicknesses. (a) Absolute E‐field distribution; (b) Corresponding E‐field gradients, where a conservative estimation for stimulation threshold can be obtained from case with a 500 µm insulating layer. The range of the ribbon ring is indicated by the grey area.

Figure 6 shows both the absolute E‐fields (Figure 6a) and the corresponding gradients (Figure 6b) for five different insulation thicknesses, in comparison to the baseline scenario. For the baseline case without a ring, the magnetic stimulation device only produces an electric field that is near constant along the nerve. In contrast, the gold ribbon ring shields the middle portion of the nerve from the E‐field whilst significantly increasing the E‐field strength at its edges, which translates into the appearance of strong gradients near both ends of the ring. As the insulation increases, the peak gradient decreases until, at the largest featured thickness of 500 µm, the absolute peak gradient falls below a value of 2 mV/mm^2^, which is unlikely to activate the nerve and can be used as a conservative threshold for nerve activation. This threshold of electric gradient for effective stimulation will be later experimentally justified. In practice, as the ribbon ring without an insulation layer will not become dislodged by more than 100 µm, neural activation is likely to occur.

Three key factors contribute to the creation of large E‐field gradients: (1) the electrical shielding provided by the ring around the middle of the nerve, which is most prominent in the scenario with no insulation; (2) the edges of the ring, which create E‐field peaks that become weaker when more separated from the nerve; (3) the interfaces in‐between the ring, the insulating layer, the nerve, and the surrounding tissue, which direct E‐fields perpendicular to the nerve in a small region, as seen in Figure 5.

### 3.3 Insulation effects: Comparison between modeling and experiments

**Figure 7:**
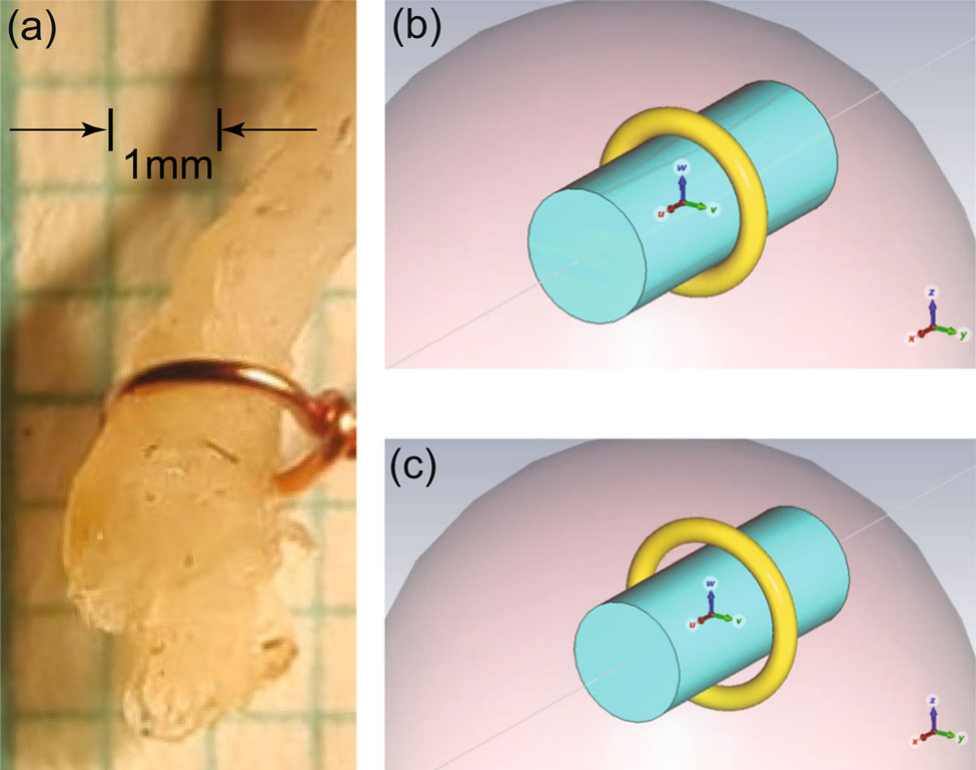
Experimental and modeled nerves wrapped by a toroidal ring. (a) Image of the sciatic nerve inside a copper loop. Graph paper with a 1 mm grid is visible in the background. The wire thickness is 100 ± 10 µm. A gap of 85–118 µm is visible between the nerve and the top ring where the wire is twisted; (b) Model of the nerve with toroidal ring in contact; (c) Model of nerve with a 100 µm gap from the toroidal ring.

To facilitate the fabrication and assembly process, we performed another physical experiment to explore the contact effects by stimulating the sciatic nerve with a copper toroidal ring as described before (Sliow et al., 2019). When the toroidal ring has a 100‐µm‐thick plastic coating, action potentials are not triggered upon magnetic stimulation. In contrast, removing the coating from the ring and bringing it into contact with the nerve allows neural activation to occur, eliciting Compound Nerve Action Potentials (CNAPs) of 0.33 ± 0.05 mV (*n* = 3). During the latter experiment, the magnetic stimulation device delivers one pulse per second to the ring and 39 times out of 120 pulses, the action potential is not generated. This can be attributed to the limb movement after a successful action potential that displaces the ring. Usually, at least one part of the ring will be in contact with the nerve, as shown in Figure 7a, but the sudden movement of the limb may create a temporary gap between the nerve and the ring, causing misfiring of the subsequent action potential.

To understand the fields being generated by the copper toroidal ring, we emulated these in vivo experiments in our computational simulations. The rectangular cross‐section of the gold ribbon ring is replaced by the circular cross‐section of a copper toroidal ring, as defined by the parameters in Table 3. The model reuses the same relative positions, dimensions, properties of the nerve, and the surrounding tissue sphere. As shown in Figure 7b,c, one configuration comes with a toroidal ring in contact with the nerve while the other has a 100 µm gap in‐between. Both cases are investigated for comparison with experiments: when the toroidal ring is in contact with the nerve and when it gets dislodged due to twitching.

**Table 3:** Properties of toroidal ring.

Parameter	Size (contact/noncontact)	Units
Inner radius	0.5/0.6	mm
Outer radius	0.6/0.7	mm
Cross‐section diameter	0.1	mm
Conductivity	5.8 × 10^7^	S/m

**Figure 8:**
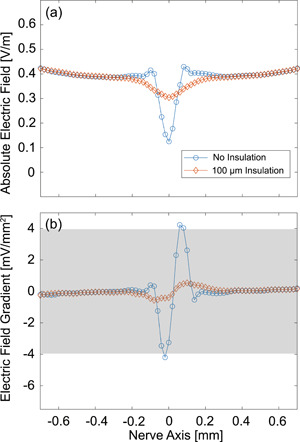
Plots of the simulated E‐field strengths and calculated gradients along the offset nerve axis 30 µm under the surface of the nerve when the toroidal ring is in‐contact and isolated. (a) Absolute E‐field distribution; (b) Corresponding E‐field gradients, where the estimated conservative stimulation threshold is indicated by the grey area.

The simulation results show that the E‐field gradients are significantly larger when the ring is in contact with the nerve as illustrated in Figure 8. Since the toroidal ring causes neural activation with no insulation, but fails when a 100 µm gap exists, the calculated gradients can be used to further justify the previously estimated stimulation threshold. The midpoint between the peak gradients of the two scenarios is 2.4 mV mm^−2^ and provides a ball‐park estimate for the lower bound of thresholds. Thresholds of 4 and 5 mV mm^−2^ were calculated by Kosta et al. (2019, 2020) respectively using axon diameters of 20 and 10 µm. In practice, rat sciatic neurons are slightly larger (mean diameter of 29 µm) and are easier to stimulate (Swett et al., 1991). Therefore, to calibrate our model with our experimental results, and to match previous literature, we will consider a range of thresholds between 2 and 4 mV mm^−2^ for being required for neural activation. Gradients in this range may be able to stimulate neurons but will be less reliable. To be specific, the peak gradients of the in‐contact case are above the upper of the threshold range whilst the peak gradients of the isolated case are below the lower boundary. Referring to the level of peak gradients in Figure 6, the observed difference in level with Figure 8 suggests that the geometry of the ring, likely the presence and sharpness of edges, is an important consideration in how reliably and efficiently neural activation will be achieved.

## 4 DISCUSSION

This paper suggests that the cause for the electrical stimulation of nerves through the use of the graft‐antenna system that was first proposed by Sliow et al (Sliow et al., 2019), is due primarily to the electric fields created at the edge of the ring which sets up high‐intensity field gradients in a small region around it. Our computational model demonstrated that direct contact between the ring and nerve ensures neural activation, in agreement with the experimental in vivo data.

A large gap of 500 µm was assumed to prevent neural activation when the gold ribbon ring of the graft‐antenna is considered, and a conservative stimulation threshold can be estimated accordingly. In another experimental validation, a gap of approximately 100 µm prevented neural activation for the toroidal ring. The simulation model is robust to variations of the surrounding tissue shape or ribbon ring thickness.

The model has assumed that activation of neurons 0.03 mm deep under the surface of the nerve was a measure of whether or not the entire nerve would elicit a response. However, the nerve bundle actually contains thousands of axons (Figure 1a) which have been subjected to E‐fields of varying magnitudes, as can be seen in Figures 4 and 5. The deeper axons may or may not be activated by the graft‐antenna, and due to heterogeneities in surrounding axons, tissue, and blood vessels, the activation distribution is unlikely to be cylindrically symmetric. If they are not activated beyond a certain depth, there is a possibility that some induced bundle effect allows the surface neurons to synchronise and induce activations in deeper neurons, though further modeling and experimentation are needed to investigate this hypothesis. The curvature of the nerve or neuronal undulations also cannot be fully captured with the simplified model.

Additionally, a finite strip of nervous tissue in a sphere of muscular tissue is an approximation of the rat sciatic nerve and surrounding tissue. In the Supplementary Section, we demonstrate that the shape of surrounding tissue and basic heterogeneities in the nerve did not qualitatively change the results of using a graft‐antenna for neural activation. However, they did cause quantitative changes which will need to be accounted for since they contribute to stimulation depth. The magnetic stimulation device was also assumed to be exactly 60 mm away from the graft antenna and directed transverse to the nerve's length. In practice, these will not be perfectly controlled since the graft‐antenna will be concealed within the subject's body and, as a result, there will be further quantitative differences between the model and experimental results.

Finally, experimental validation for the effect of insulation layers between the gold ribbon ring and the nerve is needed. Repeating the insulation experiments described in this paper with built‐in insulation layers under the ribbon ring could provide more insight into the point at which neural activation starts failing experimentally and also into the behavior of very thin (<10 µm) insulation layers where slightly stronger peak gradient is generated compared to the in‐contact case. These insights could motivate further design improvements.

## CONFLICT OF INTEREST STATEMENT

The authors declare no conflict of interest.

## ETHICS STATEMENT

L. A. S., J. D. B., C. F., and G. C. T. designed theoretical research and numerical investigations. A. L. provided the graft antennas and designed the in vivo experiments with the help of A. S., Z. M., D. A. M. L. A. S., and J. D. B. performed theoretical research and simulations. Z. M., A. S., A. L., and D. A. M. performed the experimental research. L. A. S., J. D. B., and A. L. analysed data with the supervision of C. F., G. C. T. Lastly, M. R. H. L. A. S., J. D. B., X. L., A. L., C. F., and G. C. T. wrote the paper and finalised it with the help of all the other authors.

## Supporting information

Supporting information.
