# Supplementary material for: Investigation of the mechanisms for wireless nerve stimulation without active electrodes
Source: Bioelectromagnetics. 2023 Nov 1;44(7-8):181–91. doi: 10.1002/bem.22486 (PMC10947236; doi:10.1002/bem.22486)
Supplement: Supplementary file 1 — Supporting information. [file BEM-44-181-s001.docx]

**SUPPLEMENTARY SECTION**

**Magnetic stimulation device characteristics**

There are three characteristics of importance for modelling the magnetic stimulation device. The first characteristics are its physical dimensions which are specified in [11] and summarized in Fig. 9. The inner and outer radii of each wing is 25 mm and 45 mm respectively and each wing is made of 11 concentric circles instead of the conventional single-conductor spiral wiring. However, these dimensions don’t have a significant effect on the magnetic fields at distances further than approximately 30 mm [25] and could instead be approximated by a single current loop with no extra dimensionality. The magnetic fields produced by this device along the zy-plane are shown in Fig. 1(a) of the main text. The other two characteristics are the peak current amplitude and the effective current frequency which can both be calculated from experimental data obtained using a Hall effect probe to measure the magnetic fields produced by the magnetic stimulation device.

The biphasic current which typically pulses through a magnetic stimulation de-vice can be modelled as a single damped oscillator operating at one frequency [21]. By taking the Fourier transform of the pulse measured by the Hall effect probe, the resonant frequency was found to be 3544 Hz, as shown in Fig. 10. This frequency satisfies the quasi-static approximation condition:

f << (c/d) (1)

where f is the frequency of the electro-magnetic fields, c = 3.0 × 108 ms^−1^ is the speed of light, and d < 1 m is largest length scale of the problem domain. Therefore, the Ohmic currents within the tissue dominate the displacement currents and the following equation can be used to solve for the divergence of the electric vector potential, ∇φ, within the nerve:

∇ · σ∇φ = −jω∇ · (σA_0_) (2)

Figure 9: Overhead view of the model magnetic coil.

where ∇· is the divergence operator, σ is the conductivity of the material, j = $\sqrt{-1}$ is the imaginary number, ω = 2πf is the an-gular frequency, and A_0_ is the magnetic vector potential, which is calculated from the source currents using the Biot-Savart Law [26]. From equation 2, the electric vector field magnitude, E_0_, is calculated using the relationship E_0_ = ∇φ. Further-more, the time-dependent electric field is E = E_0_ exp (jωt). Equation 2 is independent of material permittivity ($\epsilon$) and permeability (µ) and depends only on the conductivity (σ). The angular frequency is a constant scale factor throughout space and doesn’t affect the spatial distribution of the E-fields, only their magnitudes. In a similar way, the imaginary number tells us that the magnetic fields and electric fields are 90 degrees out of phase with each other but in phase with themselves at all points in space. It also implies secondary induced fields from the graft-antenna are insignificant compared to the fields produced by the magnetic stimulation device since the phase is not dependent on the existence of materials. In addition, the electric vector field alignments are independent of time, but the magnitudes oscillate from positive to negative at a fundamental frequency of 3544 Hz with additional harmonics every subsequent 3544 Hz. A summary of the magnetic coil physical characteristics used in this work is provided in Table 4.

Table 4: Summary of magnetic coil characteristic values

| **Parameter** | **Value** |
| --- | --- |
| Number of Loops | 11 |
| Inner Radius | 25 mm |
| Outer Radius | 45 mm |
| Peak Current Magnitude | 1 kA |
| Pulse Fundamental Frequency | 3544 Hz |

**Model robustness: tissue shape and ring thickness**

We take a brief aside to compare among the use of a spherical and cubical surrounding tissue, and the heterogeneous and homogeneous nerve tubes. In all cases, the graft-antenna used is the rib-bon ring in contact with the nerve. For the cubical model, the surrounding tissue cube has a side length equal to the diameter of the tissue sphere (5 mm). The simplified heterogeneous model is generated under identical conditions to the default spherical case, except that the nerve is modelled based on a histological cross-section with blood vessels and surrounded by a layer of perineurium tissue (see Fig. 2(b)). Although the shapes of the E-field gradient curves are similar, as shown in Fig. 11, the square surrounding tissue has the largest maximum gradient while the heterogeneous nerve has the widest range of gradients above thresh-old indicating more of the nerve is stimulated. As the results obtained through different software, boundary conditions, and geometries are very similar, the qualitative reliability of the model for simulating neural activation can been verified.

The ribbon ring as the graft-antenna is approximately 70 nm thick. To reduce computational complexity, however, a thicker 1 µm ribbon ring is used in the simulation model. To test the effect of this adaption, the scenario that the ribbon ring is in-contact with the nerve is simulated for five different ring thicknesses. Fig. 12 shows that the ring thickness determines how sharp the E-field peaks are at the edges, where a thinner ring generally corresponds to a sharper peak and larger peak gradient. Therefore, the calculated gradients in this paper are probably slight underestimates of the gradients generated during the physical experiments adopting a 70 nm thick ring.

Figure 10: Frequency spectrum of magnetic stimulation pulses zoomed around the first 10 kHz. The resonant frequency of the system is situated at approximately 3544 Hz as marked by the red dashed line. Inset is the time evolution of the magnetic pulses, as measured by a Hall effect probe positioned 60 mm from the magnetic coil by Sliow et al. [27]. The duration of the pulse is slightly less than 350 µs and a calibration current of 1 A is used.

Figure 11: Electric field gradients calculated based on the simulated E-field strengths of the ribbon ring along the offset nerve axis 30 µm under the surface of the nerve when applying: cubical surrounding tissue, spherical surrounding tissue, and heterogeneous nerve with spherical surrounding tissue. The cubical and spherical model data is generated with CST™. The heterogeneous nerve model data is generated with Sim4Life™.

Figure 12: Simulated E-field strengths and calculated gradients along the offset nerve axis 30 µm under the surface of the nerve when the ribbon ring is in-contact.

**Surgical procedure**

The rats were anesthetised using 2% Isofluorane in 100% oxygen, a 3 cm–4 cm skin incision was then made between the ischial tuberosity and the knee joint of the right leg, under sterile conditions. The plane between the gluteus maximus and biceps femoris muscles was identified and dissected using blunt dissection to ex-pose ≈ 1.5 cm of the sciatic nerve proximal its distal trifurcation. Using an Olympus operating microscope (1-40x magnification), the nerve was freed by dissecting surrounding connective tissue with a microscissor, care was taken to minimize nerve handling.

**Graft-antenna fabrication and laser tissue bonding**

The graft-antenna comprises a polymeric film and a thin gold strip that is plated on the film. The film is prepared following the protocol published by our group before (1). Concisely, a solution is pre-pared of medium molecular weight chitosan (598 cps viscosity, 81% deacetylation; Sigma-Aldrich, Sydney, NSW, Australia) dissolved at a concentration of 1.7%(w/v) in deionized water, containing 2% (v/v) acetic acid and 0.01% (w/v) rose ben-gal.

After stirring the solution for 14 days at room temperature (≈25 ◦C) and in the dark to avoid rose bengal photo-bleaching, the insoluble matter is removed by centrifugation (3270×g for an hour). The purified solution is then spread evenly (≈1.2 mL over ≈12 cm^2^) on a dry and sterile Perspex plate at room temperature. The solution needs to dry for 3 weeks, causing ≈90% water content evaporation, in order to form a thin film that is insoluble in water. The rose bengal-chitosan film is carefully detached from the plate and small rectangular sections ( 5x5 mm) are cut with scissors. A strip of gold is plated over the polymeric film by an Emitech K550X gold coater (Quorum Emitech, East Sus-sex, England) and the aid of a rectangular-shaped shadow mask (5x0.8 mm) made of filter paper. The chitosan film is placed underneath the shadow mask and a gold strip is deposited with a width of 0.8 ± 0.1 mm and thickness of 50 nm–80 nm. When this chitosan film is wrapped around the nerve, the gold strip becomes a ribbon ring (loop antenna) that can receive electromagnetic radiation. These fabricated graft-antennas are stored in a sterile plastic box and kept in the dark at room temperature.

After exposing the sciatic nerves, a graft-antenna measuring 5x5 mm is sterilised by dipping in 80% ethanol for 2-3 seconds. A sterile, thin plastic backboard is then positioned underneath the sciatic nerve and the graft-antenna is placed on it. The two edges of the adhesive are lifted up from the backboard with microforceps and placed around the nerve such that the gold strip forms a ring around the nerve (Fig. 2). The graft-antenna is photochemically bonded to the nerve by a 532 nm fiber-coupled laser (core diameter of 200 mm) at 250 mW in continuous wave. The graft-antenna is spot-irradiated, and each spot ( ≈ 6 mm) receives coherent light for ≈ 5 seconds. The laser irradiates the graft-antenna for a total of ≈ 133 seconds, whilst gently rotating the nerve using the backboard. The surgeon was careful not to shine light on the gold ring to avoid detrimental temperature rise. Once the plastic backboard is taken out, muscles and skin are closed using 3-0 Vicryl sutures and surgical staples, respectively. After operation, animals were kept warm with warming blanket and monitored every 10 minutes. Buprenorphine 0.05 mg kg^−1^ SC analgesia was administrated along with topical antibacterial ointment and bittering agent to the wound. Animals were re-turned to the animal facility in individual cages with no movement restriction, once fully recovered.

**Electrophysiology measures**

The CNAPs of sciatic nerves in group 1 were measured using a purpose-built AC coupled differential amplifier (100x gain, 1 Hz High pass filter) and recorded using a digital to analog converter (Model 1401, Cambridge Electronic Design, UK). The recording electrode was fixed on the sciatic nerve 1 cm away from the toroidal cop-per ring towards the foot, the reference electrode was placed in the adjacent tis-sues while the ground electrode in the skin of the rat leg. In the graft-antenna group, the CMAP of the plantaris muscle was measured placing the recording electrode in the plantaris, the reference electrode in the adjacent tissues while the ground electrode was positioned in the skin of the rat leg. The continuity of the sciatic nerve was ensured by triggering CNAPs and a visible twitch of the leg after electrical stimulation of the nerve using a commercial constant-current stimulator (pulse duration= 500 µs, amplitude = 10 µA, 1 pulse per second). After establishing the continuity of the sciatic nerve, the magnetic stimulation de-vice delivered 120 radio-wave pulses to the toroidal ring, or the graft-antenna wrapped around the nerve. Individual and aver-aged CNAP and CMAP responses were recorded using a computer program for data analysis (LabChart, Version 8.1.5).
